# Toxicological Effects of Caco-2 Cells Following Short-Term and Long-Term Exposure to Ag Nanoparticles

**DOI:** 10.3390/ijms17060974

**Published:** 2016-06-21

**Authors:** Ni Chen, Zheng-Mei Song, Huan Tang, Wen-Song Xi, Aoneng Cao, Yuanfang Liu, Haifang Wang

**Affiliations:** 1 Institute of Nanochemistry and Nanobiology, Shanghai University, Shanghai 200444, China; chenni2686@163.com (N.C.); szmeismile@126.com (Z.-M.S.); xwsgsy@126.com (W.-S.X.); ancao@shu.edu.cn (A.C.); yliu@pku.edu.cn (Y.L.); 2 Beijing National Laboratory for Molecular Sciences, College of Chemistry and Molecular Engineering, Peking University, Beijing 100871, China; huantang@pku.edu.cn

**Keywords:** Ag nanoparticle, Caco-2 cells, surface coating, long-term, low-dose, toxicity

## Abstract

Extensive utilization increases the exposure of humans to Ag nanoparticles (NPs) via the oral pathway. To comprehensively address the action of Ag NPs to the gastrointestinal systems in real situations, *i.e.*, the long-term low-dose exposure, we evaluated and compared the toxicity of three Ag NPs (20–30 nm with different surface coatings) to the human intestine cell Caco-2 after 1-day and 21-day exposures, using various biological assays. In both the short- and long-term exposures, the variety of surface coating predominated the toxicity of Ag NPs in a descending order of citrate-coated Ag NP (Ag-CIT), bare Ag NP (Ag-B), and poly (*N*-vinyl-2-pyrrolidone)-coated Ag NP (Ag-PVP). The short-term exposure induced cell growth inhibition and death. The cell viability loss appeared after cells were exposed to 0.7 μg/mL Ag-CIT, 0.9 μg/mL Ag-B or >1.0 μg/mL Ag-PVP for 24 h. The short-term and higher-dose exposure also induced reactive oxygen species (ROS) generation, mitochondrial damage, cell membrane leakage, apoptosis, and inflammation (IL-8 level). The long-term exposure only inhibited the cell proliferation. After 21-day exposure to 0.4 μg/mL Ag-CIT, the cell viability dropped to less than 50%, while cells exposed to 0.5 μg/mL Ag-PVP remained normal as the control. Generally, 0.3 μg/mL is the non-toxic dose for the long-term exposure of Caco-2 cells to Ag NPs in this study. However, cells presented inflammation after exposure to Ag NPs with the non-toxic dose in the long-term exposure.

## 1. Introduction

Nanoparticulate (colloidal) silver has been known for more than 100 years [1]. Nowadays silver nanoparticles (Ag NPs) have been used in personal care, household, medical products, and food packaging, owing to their broad-spectrum antimicrobial properties [2]. The annual production of Ag NPs is estimated to be 320 tons and is expected to grow in the coming years [1,3]. The processes, such as synthesis, handling, usage, and disposal of Ag NPs, increase the chance of human contact with the Ag NPs greatly through different pathways. Ag NPs may enter the gastrointestinal tract via contaminated water and food. In addition, it is estimated that approximately 14% of Ag nanotechnology products could release particles into the air during use [4]. The inhaled NPs trapped in the mucus of the respiratory tract can also translocate to the gastrointestinal tract [5].

Although a lot of studies have been done focusing on the toxicity of Ag NPs and Ag NPs are found toxic to different cells [6,7,8,9], there are few toxicity studies of Ag NPs to the gastrointestinal tract. In addition, the related studies evaluated the potential impact in terms of acute exposure, *i.e.*, the short-term exposures to high doses of Ag NPs [10,11,12]. However, the real exposure scenario is the long-term exposure to the low-dose of Ag NPs, which may result in different biological sequences compared to the acute exposure to the high-dose of Ag NPs [13,14,15,16]. Comfort *et al.* observed that pg/mL range of 50 nm Ag NPs did not induce a cytotoxic response, but activated sustained stress and signaling responses after the three-month incubation with keratinocytes HaCat [13]. Coccini *et al.* investigated the cytotoxicity of 20 nm Ag NPs to SH-SY5Y and D384 cells after short-term (4–48 h) and long-term (10 day) exposure [14]. They found that the toxic effects of Ag NPs started at 25 μg/mL after the short-term exposure, such as mitochondrial metabolism changes and membrane damage, while the low Ag NP doses (≤1 μg/mL) compromised the proliferative capacity of cells. Different to the intracellular production of ROS and inflammatory response caused by the short-term titanium dioxide NPs exposure, Vales *et al.* found that the long-term (three week) exposures of human bronchial epithelial cells (BEAS-2B) to low doses (<20 μg/mL) of titanium dioxide NPs did not lead to the intracellular oxidative generation, genotoxic damage, and significantly increased expression of related cytokines [15]. Different biological sequences have been observed in different cells after the short- and long-term exposure to NPs. Therefore, more attention should be paid to understand the long-term effects of Ag NPs in low concentrations and compare them with the short-term effects [13,14,15,17].

Herein, we chose Caco-2 cells as a cell model to evaluate and compare the toxicological effects of three different surface-coated Ag NPs following 1-day short-term and 21-day long-term exposure. Caco-2 cell is a cell line derived from human epithelial colorectal carcinoma cells and has been used in these experiments as the undifferentiated cell. Caco-2 cells are a general in vitro model for intestinal cells, and have been adopted by other researchers for exploring the toxicity of NPs to the gastrointestinal system [18,19,20], such short-term and long-term exposure study is valuable to draw plausible conclusions about the potential human health risk of Ag NPs exposure.

## 2. Results

### 2.1. Characterization of Ag NPs

**Figure 1:**
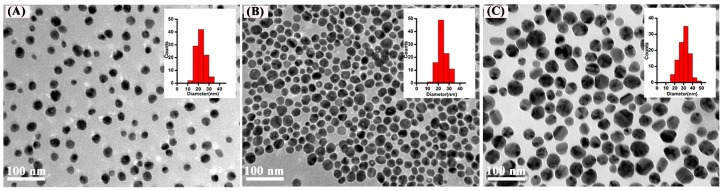
Representative TEM images of Ag NPs. (**A**) Ag-B; (**B**) Ag-CIT; and (**C**) Ag-PVP. The insert in each image shows the size distribution of the corresponding Ag NPs.

Three kinds of Ag NPs were used in this study: bare Ag NPs (denoted as Ag-B), citrate-coated Ag NPs (denoted as Ag-CIT), and poly (*N*-vinyl-2-pyrrolidone)-coated Ag NPs (denoted as Ag-PVP). They were well characterized before the biological tests. Their UV-VIS spectra display the characteristic absorption band of Ag NPs at 410 nm (Figure S1 in Supplementary Material (SM)). The PVP content in Ag-PVP NPs is 2.18% according to the thermogravimetric analysis (Figure S2 in SM). All Ag NPs are spherical and well-dispersed as shown in Figure 1. The average diameter of Ag-B, Ag-CIT and Ag-PVP is 23 ± 4 nm, 24 ± 4 nm, and 30 ± 6 nm, respectively. In aqueous solutions, Ag NPs slightly aggregate (Table 1). The hydrodynamic sizes of all Ag NPs are within 50–80 nm, about twice their primary size after 24 h incubation. After 48 h in culture medium, the size increases to 138 nm and 93 nm for Ag-CIT and Ag-PVP, respectively. The hydrodynamic size of Ag NPs is not influenced by the culture medium, but the culture time (Table 1). The ζ-potentials of Ag NPs are all negative in culture medium and change a little to more negative along time (Table 1). After 24 h in culture medium, the ζ-potentials of three Ag NPs are very similar. The increased culture time moves the ζ-potentials of Ag-CIT and Ag-PVP to more negative values.

### 2.2. Cytotoxicity of Ag NPs after the Short-Term Exposure to Ag NPs

Generally, all Ag NPs induced the viability loss and LDH leakage in Caco-2 cells in a dose- and Ag NPs sample-dependent manner.

**Figure 2:**
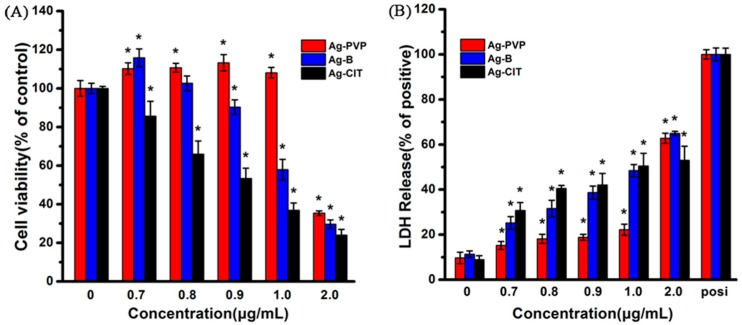
The viability (**A**) and LDH level (**B**) assays of Caco-2 cells after being exposed to three Ag NPs for 24 h. (**A**) Percent viable cells compared to no particle (0 μg/mL) condition; and (**B**) percent LDH release compared to 100% cell lysis. All data are represented as the mean ± SD (*n* = 6). * *p* < 0.05 comparing with the 0 μg/mL control.

Along with the increase of the concentration of Ag-PVP and Ag-B, the cellular viability firstly increases, and then decreases (Figure 2A). That the low concentrations of Ag NPs show the ability to stimulate the cell growth has been reported before [21]. For the Ag-CIT NPs treated cells, their viability decreases along with increase of the concentration. To make sure the coating materials of Ag NPs are safe in this study, we also tested the cytotoxicity of PVP and citrate. The content of PVP and citrate in Ag-PVP and Ag-CIT is 2.18% and less than 1.0%, respectively. At the highest concentration of Ag NPs used in this study (2.0 μg/mL), the corresponding concentration of PVP and citrate is 1.45 nM and 68.0 nM, respectively. As shown in Figure S3 in SM, the cell viabilities are higher than 90% even after cells have been exposed to PVP and citrate with a concentration up to 100 times of 1.45 nM and 68.0 nM, respectively. This indicates that the coating materials at the concentrations we used are non-toxic to Caco-2 cells.

Among three Ag NPs, Ag-CIT shows the highest toxicity and Ag-PVP the lowest toxicity. At 0.8 μg/mL, Ag-PVP increases the cellular viability to over 110%, Ag-B does not influence the cellular viability, but the viability drops to 65% after cells were exposed to Ag-CIT. When the concentration increases to 1.0 μg/mL, Ag-PVP still stimulates the cell growth, but Ag-B and Ag-CIT lead to the viability drop to less than 58% and 37%, respectively.

Many reports has attributed the dissolution of Ag NPs to the toxicity mechanism [22]. To address this clearly in this study, firstly we measured the dissolution of Ag NPs after 24 h exposure under the cell culture conditions. Only less than 3% Ag dissolved (Figure S4 in SM). Then we measured the viability of cells exposed to Ag ions. It is found that there is no viability loss when cells are exposed to 0.7 μg/mL Ag ions (Figure S5 in SM). In addition, we incubated Ag NPs in the cell culture medium for 24 h, collected the culture medium by centrifugation (to remove Ag NPs), cultured cells using the collected culture medium for 24 h, and measured the viability of cells using the CCK-8 kit. The results (Figure S6 in SM) show that collected culture medium, which contains the dissolved Ag from Ag NPs, do not induce any viability loss after 24 h culture. All of these results suggest that the dissolved Ag does not induce the toxic effects under the condition in this study.

Usually, the death of cells is accompanied with cell membrane damage, which could be reflected with the LDH leakage from cells. As shown in Figure 2B, the LDH levels of all Ag NPs increase with an Ag NPs sample- and concentration-dependent manner, consisting with the cell viability measurements. The cell damage induced by Ag NPs presents an increasing trend starting from Ag-PVP to Ag-B and Ag-CIT.

**Figure 3:**
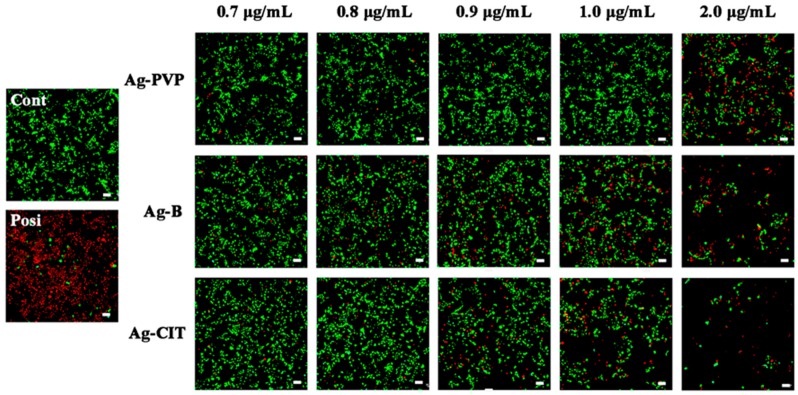
The representative live/dead staining images of Caco-2 cells after being exposed to three Ag NPs with different concentrations for 24 h. The positive control was prepared by treating normal cells with 200 mM H_2_O_2_ for 10 min. Scale bar: 60 μm.

In order to confirm the cell death induced by Ag NPs, the live/dead assay was performed to display the situation directly. The representative results are shown in Figure 3, in which green represents live cells and red represents dead cells. Along with the concentration increase, increased dead cells and decreased cell density are observed. Among three Ag NPs, Ag-CIT and Ag-B show more serious damage to Caco-2 cells. At 1.0 μg/mL, no distinguished cell death and cell density decrease is found in the Ag-PVP treated cells; however, these are significant in the Ag-B- and Ag-CIT-treated cells. Such phenomenon becomes more distinguished at 2.0 μg/mL of Ag NPs. Once again, we prove that cytotoxicity of Ag NPs mainly relies on the concentration and surface property of Ag NPs. In addition, Ag NPs not only kill cells, but also inhibit the cell proliferation.

### 2.3. Apoptosis of Caco-2 Cells after the Short-Term Exposure to Ag NPs

**Figure 4:**
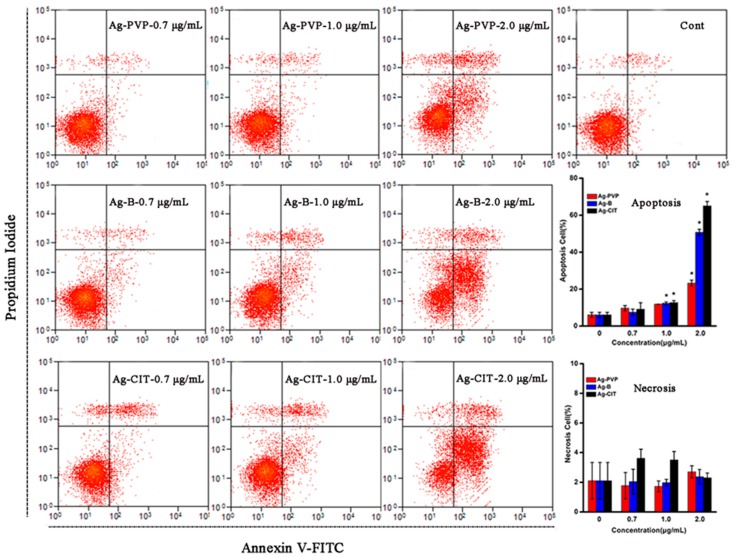
FACS scatter diagrams of Caco-2 cells by the Annexin V-FITC and PI assay. Caco-2 cells had been exposed to three Ag NPs with different concentrations for 24 h. For the histograms, all data are represented as the mean ± SD (*n* = 3). * *p* < 0.05 comparing with the 0 μg/mL control.

We used Annexin V-fluorescein and propidium iodide (PI) cell staining to investigate whether Ag NPs caused apoptosis. As shown in Figure 4, Ag NPs begin to induce serious apoptosis of Caco-2 cells at concentrations of 1.0 μg/mL. Apoptosis caused by Ag-CIT and Ag-B is more significant than that by Ag-PVP, which is consistent with the results of the CCK-8, LDH leakage and live/dead staining assays.

### 2.4. MMP and ROS Assays of Caco-2 Cells after the Short-Term Exposure to Ag NPs

**Figure 5:**
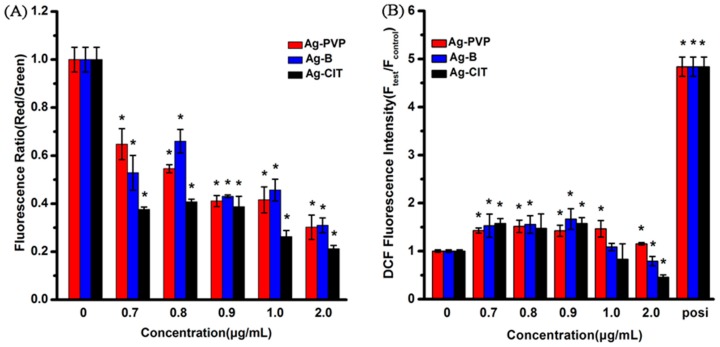
The mitochondrial membrane potential (MMP) (**A**) and ROS levels (**B**) of Caco-2 cells after being exposed to Ag NPs for 24 h. The positive control was prepared by culturing normal cells in medium containing 10 μg/mL Rosup for 30 min. All data are represented as the mean ± SD (*n* = 6). * *p* < 0.05 comparing with the 0 μg/mL control.

The decreased MMP (mitochondrial membrane potential) can be used as an early marker of the onset of apoptosis [23]. The effect of Ag NPs on the MMP of Caco-2 cells was measured using the JC-1 staining. JC-1 exists either as a green fluorescent monomer at depolarized membrane potentials or as a red fluorescent aggregate at hyperpolarized membrane potentials. The change of MMP is expressed as the fluorescence intensity ratio of red/green. The results (Figure 5A) show that the higher concentration of Ag NPs is adopted, the more cells exhibit a depolarized MMP, which indicates the mitochondrial damage. When cells have been incubated with 1.0 μg/mL Ag-CIT for 24 h, the fluorescence intensity ratio drops from 1, for the control group, to 0.3. Compared with Ag-CIT, Ag-B and Ag-PVP display less disturbance to the MMP of cells. Similar to the above assays, Ag NPs cause the MMP changes in Caco-2 cells with a concentration- and sample-dependent profile.

ROS generation is one commonly proposed toxicological mechanism of NPs [23]. The MMP decrease has a close relationship with the ROS generation [24]. As shown in Figure 5B, compared with the control group, an enhanced ROS level is detected in all Ag NPs-treated cells at lower concentrations. At higher concentrations of Ag NPs, the ROS levels decrease, especially for the Ag-CIT treated cells. The reason leads to this phenomenon is the significant cell death and density decrease at higher concentrations of Ag NPs. The similar phenomenon has been reported in Caco-2 cells exposed to ZnO NPs [10]. Contrary to the above assays, the ROS levels are similar among three Ag NPs. The decreased MMP and the up-regulated intracellular ROS level along with increased concentrations of Ag NPs refer that Ag NPs exhibits their toxicity through the intrinsic ROS-mediated mitochondrial pathway.

### 2.5. Cell Cycle Assay of Caco-2 Cells after the Short-Term Exposure to Ag NPs

**Figure 6:**
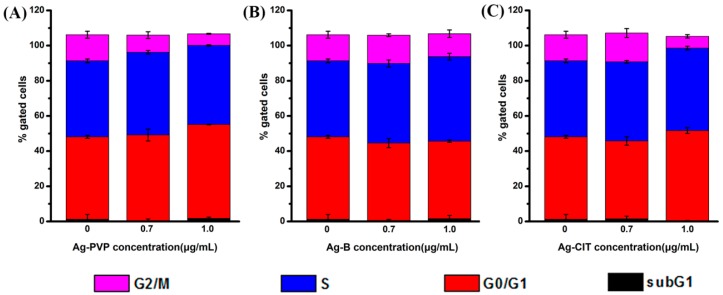
Cell cycle distribution of Caco-2 cells after exposed to three Ag NPs for 24 h. (**A**) Ag-PVP NPs; (**B**) Ag-B NPs; (**C**) Ag-CIT NPs. All data are represented as the mean ± SD (*n* = 3).

The cell cycle consists of interphases (G1, S, and G2) and mitosis (M). During the G1 phase, the cells increase in size, produce RNA, and synthesize proteins for DNA formation. DNA replication occurs at the S phase and the cells continue to grow, producing new proteins at the G2 phase. Cell division completes at the M phase. The proportion of cells in each phase was determined after 24 h of the Ag NPs treatment. The proportion of cells in the G2/M period decreased in a dose-dependent manner after cells were exposed to Ag NPs (Figure 6). When the concentration of Ag NPs increases to 1.0 μg/mL, a small amount of cells accumulate in the sub-G1 phase, which indicates the apoptosis of cells [25,26].

### 2.6. Cytokine IL-8 Secretion of Caco-2 Cells after the Short-Term Exposure to Ag NPs

**Figure 7:**
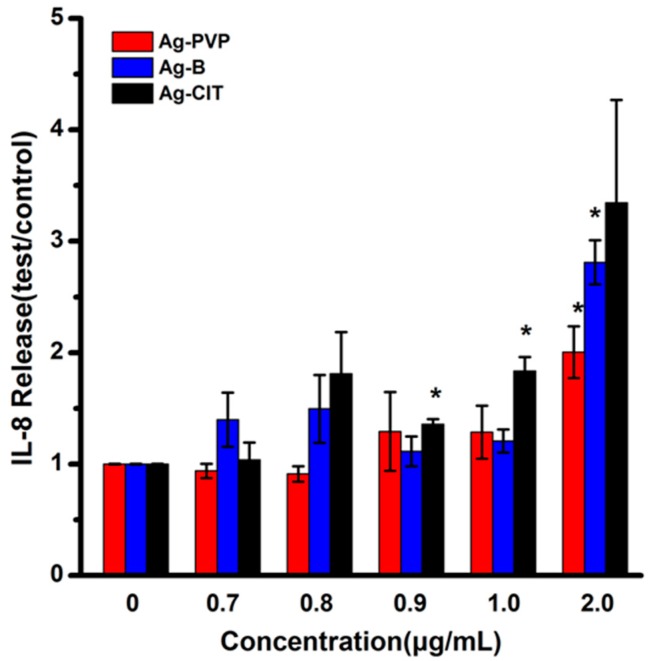
IL-8 secretion of Caco-2 cells after exposed to Ag-PVP, Ag-B, and Ag-CIT for 24 h. All data are represented as the mean ± SD (*n* = 6). * *p* < 0.05 comparing with the 0 μg/mL control.

To investigate whether the short-term exposure of Caco-2 cells to three Ag NPs causes the inflammatory response, the IL-8 secretion of Caco-2 cells was analyzed after the 24 h exposure. The results in Figure 7 illustrate that three Ag NPs do not increase the IL-8 level when the cell viabilities remain normal. Ag-CIT at 0.9 μg/mL begins to increase the IL-8 secretion, while Ag-CIT and Ag-B just increase the IL-8 secretion when the concentration is 2 μg/mL.

### 2.7. Proliferation of Caco-2 Cells after the Long-Term Exposure to Ag NPs

**Figure 8:**
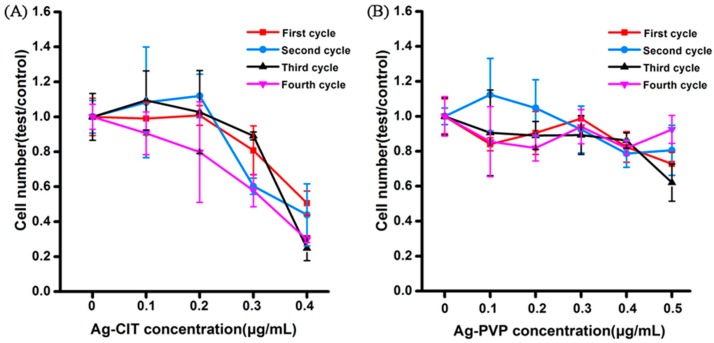
Proliferation of Caco-2 cells after exposed to three Ag NPs. Cells had been repeatedly exposed to Ag-CIT and Ag-PVP for 4 cycles and counted after each exposure circle, along with corresponding untreated controls. (**A**) Ag-CIT; (**B**) Ag-PVP. Three parallel well were set for each sample and cells in each well were counted three times.

In the short-term exposure experiments, the cytotoxicity of Ag-B falls between Ag-PVP and Ag-CIT, but more closely to Ag-CIT. Ag-B and Ag-CIT display very similar hydrodynamic size, ζ-potential, cell viability, cell apoptosis *etc.* Considering the comparable short-term cytotoxicity of Ag-CIT and Ag-B, only Ag-CIT and Ag-PVP were selected for the long-term toxicity study. The cell proliferation is a direct indicator of cell damage. We counted the cell number after each exposure cycle (five days) to evaluate the damage of cells induced by Ag NPs (Figure 8). Ag-CIT and Ag-PVP display much different influence on the cell proliferation. At concentrations up to 0.5 μg/mL, Ag-PVP does not affect the cell growth, even after four exposure cycles, that is, 21 days. However, Ag-CIT begins to inhibit the cell proliferation at 0.3 μg/mL. At 0.3 μg/mL of Ag-CIT, the obvious cell growth inhibition is observed even after the first exposure cycle. When the concentration increases to 0.4 μg/mL, only about 20% cells survive after the forth cycle. Consistent with the short-term results, Ag-CIT displays much higher toxicity than Ag-PVP. The results demonstrate that it is non-toxic for the long-term exposure of Caco-2 cells to Ag NPs less than 0.3 μg/mL.

### 2.8. Viability and Membrane Integrity of Caco-2 Cells after the Long-Term Exposure to Ag NPs

**Figure 9:**
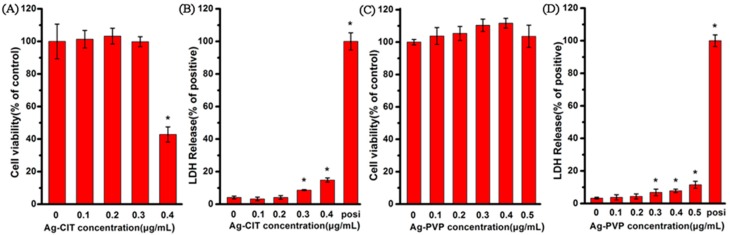
The viability assay and LDH levels of Caco-2 cells after exposed to Ag-CIT (**A**,**B**) and Ag-PVP (**C**,**D**) for 21 days. (**A**,**C**) Percent viable cells compared to the no particle (0 μg/mL) condition. (**B**,**D**) Percent LDH release compared to 100% cell lysis. All data are represented as the mean ± SD (*n* = 6). * *p* < 0.05 comparing with the 0 μg/mL control.

After Caco-2 cells had been exposed to Ag-CIT and Ag-PVP for four cycles, the cell viability and the released LDH level were measured. As shown in Figure 9, there is no viability loss after cells exposed to Ag-CIT and Ag-PVP for 21 days, except Ag-CIT at 0.4 μg/mL. The cellular viability drops to less than 50% after cells have been exposed to 0.4 μg/mL Ag-CIT for 21 days. According to the cell viabilities, we define the non-cytotoxic dose of Ag NPs at 0.3 μg/mL and lower [27].

When the concentration of two Ag NPs reaches 0.3 μg/mL, the LDH level begin to increase significantly compared with the control, however, the highest value is still less than 15% of the positive control. This suggests that the cell integrity damage is a more sensitive parameter than the viability loss. Consistent with the viability assay, 0.4 μg/mL Ag-CIT induces the highest LDH release from cells.

### 2.9. Live/Dead Staining of Caco-2 Cells after the Long-Term Exposure to Ag NPs

**Figure 10:**
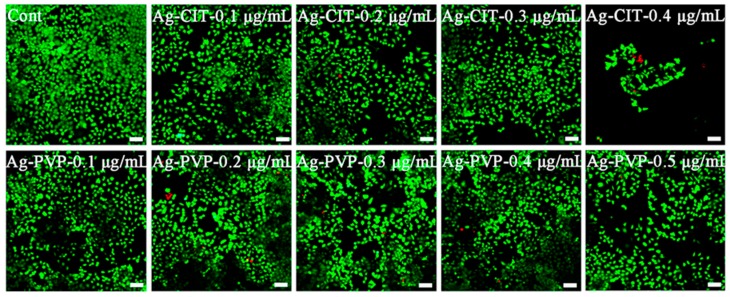
The representative live/dead staining images of Caco-2 cells after exposed to Ag-CIT and Ag-PVP in different concentrations for 21 days. The positive control was prepared by treating normal cells with 200 mM H_2_O_2_ for 10 min. Scale bar: 60 μm.

To confirm the results obtained from the CCK-8 and LDH assays, we imaged the live and dead cells under the microscope after staining. In Figure 10, we can hardly find dead cells, even after being exposed to 0.4 μg/mL Ag-CIT. The only change, a significant cell density drop, is found in cells exposed to 0.4 μg/mL Ag-CIT. While the cell density still remains normal in cells exposed to 0.5 μg/mL Ag-PVP. The results match perfectly with those obtained from the viability assays (Figure 9A,C). Generally, Caco-2 cells are tolerant to Ag NPs with a concentration up to 0.3 μg/mL; Ag-CIT at higher concentrations mainly inhibit the cell growth.

### 2.10. Nucleus Changes of Caco-2 Cells after the Long-Term Exposure to Ag NPs

The toxic effect of cells may also be reflected by the morphology of nucleus. We, therefore, investigated the cells using the optical microscope after the DAPI staining.

**Figure 11:**
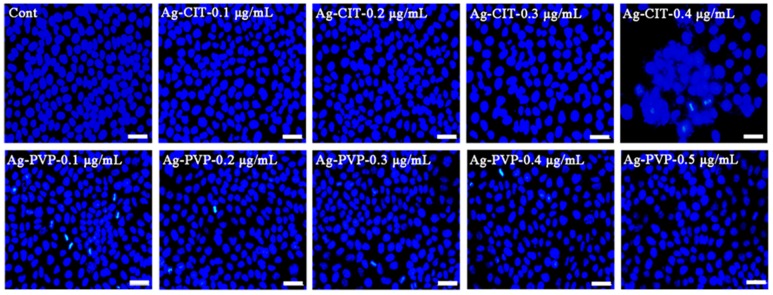
The representative pictures of Caco-2 cells exposed to Ag NPs for 21 days. Cells were stained with DAPI and investigated under the fluorescence microscope. Scale bar: 30 μm.

Figure 11 shows microscopic pictures of Caco-2 cells have been exposed to Ag-CIT and Ag-PVP for 4 exposure cycles. We observe that the cell nuclei are normal even though the cells have been exposed to 0.3 μg/mL Ag-CIT or 0.5 μg/mL Ag-PVP. However, the cell nuclei swell and fragment in cells exposed to 0.4 μg/mL Ag-CIT; cells treated with 0.5 μg/mL Ag-PVP show a slightly decreased cell density. The images of the nucleus staining is consistent with those of the live/dead staining assay (Figure 10).

### 2.11. Cytokine IL-8 Secretion of Caco-2 Cells after the Long-Term Exposure to Ag NPs

**Figure 12:**
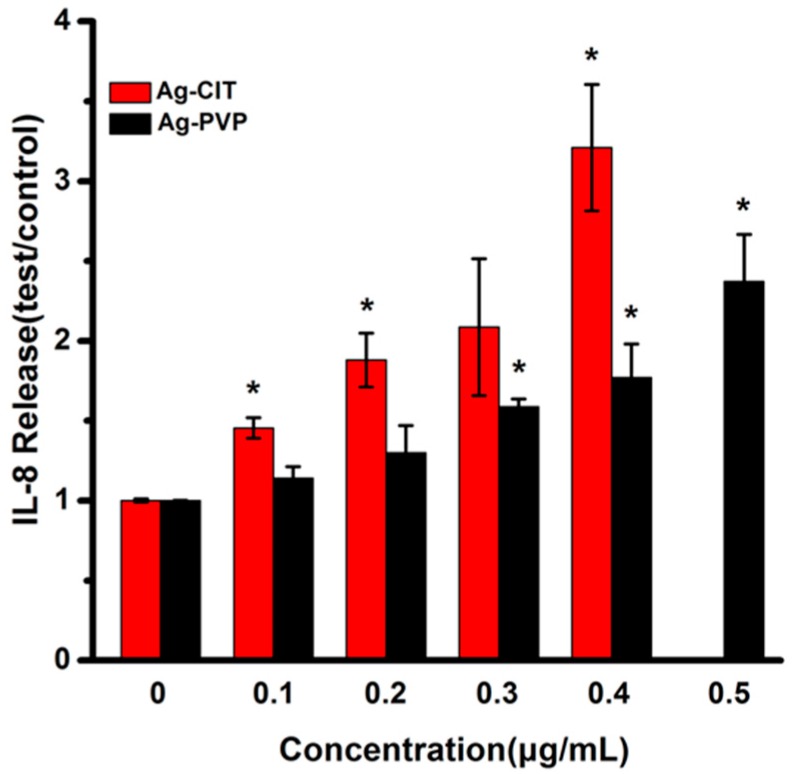
IL-8 secretion of Caco-2 cells after the long-term exposure to Ag-CIT and Ag-PVP for 21 days. All data are represented as the mean ± SD (*n* = 6). * *p* < 0.05 comparing with the 0 μg/mL control.

To investigate whether long-term exposure of Caco-2 cells to Ag NPs causes the inflammatory response, the IL-8 secretion of Caco-2 cells was analyzed after the 21-day exposure. The data in Figure 12 illustrate that both Ag-CIT and Ag-PVP increase the IL-8 level with a concentration-dependent profile, while Ag-CIT has the higher ability inducing the IL-8 secretion. Different with other assays, Ag-CIT at 0.1 μg/mL increases the IL-8 secretion. For Ag-PVP, the minimal concentration for the increased IL-8 secretion is 0.3 μg/mL. Clearly, Ag-CIT is easier to induce the inflammation in cells than Ag-PVP.

## 3. Discussion

Due to their excellent antimicrobial properties, Ag NPs have been widespread use in various fields, and have become the most commonly used engineered NPs in commercial products [28]. Hence, the exposure of humans via different pathways to Ag NPs is inevitable. The oral exposure is one major route with the feature of the low-dose and long-term. Although the vast toxicological data about Ag NPs are available [22], most of them were obtained by the short-term exposure, which may not reflect the real situation of human exposure. Therefore, in this study, we evaluated the cytotoxicity of Ag NPs after the long-term exposure and compared the results with those by the short-term exposure.

Although we found that both the short- and long-term exposures to Ag NPs induced the cell proliferation inhibition, the significant difference was also observed. The marked cell death was observed after the short-term exposure (Figure 3), but not the long-term exposure (Figure 9). In addition, the cell viability loss appeared after cells were exposed to 0.7 μg/mL Ag-CIT, 0.9 μg/mL Ag-B or >1.0 μg/mL Ag-PVP for 24 h. However, after 21-day exposure to 0.4 μg/mL Ag-CIT the cell viability dropped down to less than 50% and cells exposed to 0.5 μg/mL Ag-PVP kept normal as the control. The short-term higher-dose exposure also induced ROS generation, mitochondrial damage, cell membrane leakage, apoptosis, and inflammation (IL-8 level). The long-term lower-dose exposure induced the inflammation. Generally, 0.3 μg/mL was the non-toxic dose for the long-term exposure of Caco-2 cells to Ag NPs in this study. The different short- and long-term biological effects of Ag NPs and other NPs have been investigated in other cells and the bioeffects also are dependent on the cell lines [13,14,15].

Surface property is a critical factor influencing the biosequence of NPs in biological systems [29,30,31]. In this study, except bare Ag NPs, two commonly and widely used surface coatings, PVP and citrate, were selected. Unsurprisingly, we observed a distinct toxic difference among three Ag NPs samples. Ag-CIT shows the highest toxicity, followed by Ag-B, and Ag-PVP displays the lowest toxicity. The difference may come from different protein corona on the surface of Ag NPs, which not only changes the interaction of Ag NPs and cells, but also changes the dissolution of Ag NPs in systems [22]. Interestingly, three Ag NPs show the same order of toxicity in both the short- and long-term exposure. We may evaluate the toxic trend of different Ag NPs by using the short-term exposure.

Although the dissolution of Ag NPs is thought to be main toxic mechanism by many researchers [22], we did not find the distinct toxic effect from the dissolution of Ag NPs. We incubated Ag NPs at different concentrations with cell culture medium for 24 h. Then the culture medium containing dissolved Ag was collected by centrifuging and used to culture Caco-2 cells for 24 h. The cells grew normally without any viability loss (Figure S6 in SM). Combined with data of the dissolution of Ag NPs and the cell viability induced by Ag ions, we conclude that the dissolved Ag from Ag NPs did not damage cells under the current study conditions. Therefore, the short-term toxicity of Ag NPs comes mainly from NPs *per se*. Researchers also reported that the release fraction of several Ag NPs in cell medium did not induce any cytotoxicity and the intracellular Ag release was responsible for the toxicity [9,31].

Similar to other studies on Ag NPs [32,33,34], ROS generation was observed in this study. ROS formation and subsequent oxidative stress have been reported to be early cellular responses to NPs and play a key role in cytotoxicity [9], such as induce cell death by either apoptosis or necrosis [35,36]. This is in accordance with the data in our study. After the short-time exposure, the intracellular ROS increase was observed. Subsequently, the MMP decrease, cell cycle inhibition, and apoptosis were detected. These indicate that toxicity is the intrinsic ROS-mediated mitochondrial pathway.

In addition, IL-8 secretion was observed at cytotoxic concentrations of Ag NPs, indicating the inflammation response is consistent with the cell viability change. However, for the long-term exposure, Ag NPs trigger the release of IL-8 in Caco-2 cells at the non-cytotoxic concentrations, with a concentration-dependent style. The IL-8 is known to be secreted by epithelial cells, and is involved in the innate immune response [37]. Therefore, the immune response is an earlier effect than the viability change after the long-term low-dose exposure of Caco-2 cells to Ag NPs. More biological assays will be performed to find more sensitive biological indicators for the long-term exposure.

More Ag NPs will be produced and applied in more fields in the future; therefore, their safety assessment deserves more attentions. Published data and our study show that the long-term exposure does induce various biological responses in cells and there are distinct differences between the toxicological response of the short-term and long-term exposure. To copy the real exposure environment, the long-term and low-dose toxicity studies are essential in the future. According to our results, the long-term exposure usually does not show obvious cell inhibition and death, thus more sensitive biological indicators or molecules, such as IL-8 in this study, should be tested to reveal the earlier change induced by Ag NPs.

## 4. Experimental Section

### 4.1. Ag NPs and Their Physicochemical Characterization

Three kinds of Ag NPs were used in this study. Citrate-coated 25 nm Ag NPs (denoted as Ag-CIT, the citrate content less than 1.0%) and bare 25 nm Ag NPs (denoted as Ag-B) were purchased from Beijing DK Nano Technology Co., Ltd. (Beijing, China). Poly (*N*-vinyl-2-pyrrolidone)-coated Ag NPs (denoted as Ag-PVP) were synthesized following the previous report [38].

The size and morphology of Ag NPs were characterized with a JEM-200CX transmission electron microscope (TEM, JEOL, Tokyo, Japan). The size distribution was obtained by measuring at least 100 particles in TEM images using ImageJ software (National Institutes of Health, Bethesda, MD, USA). The UV–vis spectra of Ag NPs were recorded by a UV–VIS spectrophotometer (Lambda 35, Perkin Elmer, Norwalk, CT, USA). The PVP content of Ag-PVP NPs was determined by thermogravimetric analysis (TGA, Q600 SDT, TA instruments, New Castle, DE, USA).

The average hydrodynamic size and ζ-potential of Ag NPs in aqueous solutions were determined by a nanosizer (Nano ZS90, Malvern Instruments, Malvern, Worcus, UK). Briefly, Ag NPs were suspended in water and DMEM medium containing 10% fetal bovine serum (FBS). The Ag NPs suspensions were kept in the incubator (37 °C, 5% CO_2_/95% air) for 0–48 h and then measured. The pH value of the medium was kept around 7.4 during the incubation process.

The dissolution of Ag NPs were measured by inductively-coupled plasma mass spectrometry (ICP-MS, PerkinElmer, Norwalk, CT, USA). Ag NPs suspended in DMEM medium containing 10% fetal bovine serum (FBS) and cultured in incubator at 37 °C a humidified atmosphere of 5% CO_2_/95% air for 24 h. The suspensions were subsequently centrifuged (100,000× *g* for 15 min). The supernatants were collected and digested by nitric acid and hydrogen peroxide in a microwave digestion system (CEM, Mars, Matthews, NC, USA). The digested solutions were adjusted to 2% nitric acid solution and measured on the ICP-MS.

### 4.2. Cell Lines and Cell Culture

The human colon colorectal adenocarcinoma Caco-2 cell (ATCC No.: TCHu146) was obtained from the Cell Bank of Type Culture Collection of Chinese Academy of Sciences (Shanghai, China). Caco-2 cells were maintained in high glucose DMEM (4.5 g/L glucose) supplemented with 10% FBS (Sigma, Carlsbad, CA, USA), 1% non-necessary amino acid (Sigma, USA), and 1% penicillin/streptomycin. Cells were cultured at 37 °C in a humidified atmosphere of 5% CO_2_/95% air.

### 4.3. Viability and Membrane Integrity of Caco-2 Cells after the Short-Term Exposure to Ag NPs

To measure the metabolic activity and membrane integrity of Ag NPs-treated cells, we used a WST-8 cell counting kit (CCK-8, Dojindo Molecular Technologies Inc., Kumamoto, Japan) and LDH test kit (CytoTox 96^®^ Non-Radioactive Cytotoxicity Assay, Promega Corp., Madison, WI, USA), respectively. Caco-2 cells (4000 per well) in 100 µL culture medium were plated in wells of 96-well plates (Greiner Bio One GmbH, Frickenhausen, Germany) and grown overnight. Then, the medium was removed and 200 µL fresh culture medium containing different Ag NPs and corresponding coating materials (PVP and citrate) was introduced to cells. After 24 h, the culture medium was collected for the LDH assay by centrifugation (4500× *g* for 10 min) and cells for the viability assay following the procedures reported before [39].

After cells were washed with Hank’s balanced salt solution (HBSS) (Gibco, Gaithersburg, MD, USA), CCK-8 solution (100 μL, containing 10% CCK-8) was added and incubated for 1 h at 37 °C. Then, the optical density (OD) of each well at 450 nm was recorded on a microplate reader (Thermo, Varioskan Flash, Waltham, MA, USA). The cell viability (% of control) is expressed as the percentage of (OD_test_ − OD_blank_)/(OD_control_ − OD_blank_), where OD_test_ is OD of cells exposed to Ag NPs, OD_control_ is OD of the control and OD_blank_ is OD of the well without cells.

For the LDH assay, fifty microliter culture medium obtained above was taken and assayed following the instructions of the kit. The positive control was prepared by adding 10 μL lysis solution to the control cells at 45 min prior to centrifugation (4500× *g* for 10 min). The OD of each well at 490 nm was recorded on the microplate reader. LDH release (% of positive control) is presented as the percentage of (OD_test_ − OD_blank_)/(OD_positive_ − OD_blank_), where OD_test_ is the OD of control cells or cells exposed to Ag NPs, OD_positive_ is OD of positive control cells and OD_blank_ is the OD of well without cells.

Ag NPs with different concentrations were incubated with culture medium for 24 h at 37 °C in a humidified atmosphere of 5% CO_2_/95% air. Then the supernatant culture medium contained dissolved Ag was collected by centrifuging at 100,000× *g* for 15 min. The supernatant was used to culture Caco-2 cells for 24 h and the cell viability was tested after that following the method described above.

### 4.4. Live/Dead Staining of Caco-2 Cells after the Short-Term Exposure to Ag NPs

The live/dead staining kit (L-3224, Invitrogen, Carlsbad, CA, USA) was used to image the Ag NPs-treated cells. Calcein AM and ethidium homodimer-1 mix in the kit can differentiate live (green, Ex 495 nm; Em 515 nm) cells from dead (red, Ex 528 nm; Em 617 nm) cells. Cells were plated and treated with Ag NPs as described in the cell viability assay. After 24 h incubation, the culture medium was removed. The dyes dissolved in HBSS were introduced to cells and incubated for 30 min at room temperature. After that, cells were investigated under a fluorescence microscope (DMI3000, Leica, Wetzlar, Germany). As the positive control, a subset of wells were treated with 200 mM H_2_O_2_ for 10 min.

### 4.5. Apoptosis Analysis of Caco-2 Cells after the Short-Term Exposure to Ag NPs

An apoptosis kit (FITC Annexin V Apoptosis Detection Kit I, BD Biosciences, San Diego, CA, USA) was employed to detect apoptotic and necrotic cells. Caco-2 cells (1.2 × 10^5^ per well) was seeded into 6-well plates, treated with Ag NPs for 24 h, and harvested with trypsin. The cells were collected, washed twice with cold HBSS, and re-suspended in binding buffer (1 × 10^6^ cells/mL). One hundred microliters of cells were transferred to a tube, then 5 μL of FITC-conjugated Annexin V (Annexin V-FITC) and 5 μL of propidium iodide (PI) were added followed by incubation for 15 min at room temperature in the dark. The stained cells diluted by the binding buffer were analyzed by the fluorescence-activated cell sorting method (FACS, MoFlo XDP, Beckman Coulter, Fullerton, CA, USA). The positive control was prepared by culturing the control cells in medium containing 200 mM H_2_O_2_ for 30 min. The measurement showed that around 70% of cells are apoptotic cells, validating the apoptosis assay.

### 4.6. Measurement of the Mitochondrial Membrane Potential (MMP) of Caco-2 Cells after the Short-Term Exposure to Ag NPs

MMP was analyzed by the fluorescent dye JC-1 (5,5′,6,6′-Tetrachloro-1,1′,3,3′-tetraethyl-imidacarbocyanine, Beyotime Institute of Biotechnology, Nanjing, China). JC-1 is capable of selectively entering mitochondria where it forms monomers and emits green fluorescence when MMP is relatively low, or aggregates and emits a red fluorescence when MMP is high. Caco-2 cells were plated in six-well plates (1.2 × 10^5^ cells per well) and cultured overnight. Then, Ag NPs were introduced to cells and incubated for 24 h. After that, the culture medium of each well was replaced by 1 mL of fresh culture medium containing 10 μM JC-1 and the cells were incubated for 20 min in the dark. Finally, the cells were washed twice with cold staining buffer and their fluorescence intensity was monitored by the microplate reader. The MMP level (fluorescence ratio (red/green)) is expressed as the ratio of (F_test (red)_ − F_blank (red)_)/(F_test (green)_ − F_blank (green)_), where F_test_ is the fluorescence intensity of the tested wells, F_blank_ is the fluorescence intensity of the wells without cells.

### 4.7. Measurement of Intracellular ROS of Caco-2 Cells after the Short-Term Exposure to Ag NPs

The intracellular ROS level was detected using 2′,7′-dichlorofluorescin diacetate (DCFH-DA, Sigma, St. Louis, MO, USA), which could enter cells and be hydrolyzed into a fluorescent 2’,7’-dichlorofluorescin (DCFH) probe. After seeded into six-well plates (1.2 × 10^5^ per well) and grown overnight, Caco-2 cells was cultured in the fresh culture medium containing Ag NPs with predetermined concentrations. The positive controls were prepared with control cells cultured in medium containing 10 μg/mL Rosup for 30 min prior to the addition of DCFH-DA. At predetermined time points, cells were washed twice with HBSS and then 100 μL new culture medium containing 20 μM DCFH-DA was added. Thirty minutes later, the cells were washed with HBSS for three times and then soaked in 100 μL culture medium for the fluorescence intensity measurement on the microplate reader (EX485 nm/EM528 nm). ROS level is presented as the ratio of (F_test_ − F_blank_)/(F_control_ − F_blank_), where F_test_ is fluorescence intensity of cells exposed to Ag NPs or the positive control, F_control_ is fluorescence intensity of the control and F_blank_ is fluorescence intensity of well without cells.

### 4.8. Cell Cycle Analysis of Caco-2 Cells after the Short-Term Exposure to Ag NPs

The cell cycle was investigated by analyzing DNA content using flow cytometry. Caco-2 cells (1.2 × 10^5^ per well) was plated in six-well plates and incubated overnight. Then cells were exposed to Ag NPs for 24 h. After that, the cells were washed with HBSS, collected and fixed with cold 75% ethanol for 4 h. The fixed cells were washed with HBSS and then re-suspended in 100 μL RNase A. After incubated at 37 °C for 10 min, 200 μL of PI was added to cells and then the mixture was incubated at 4 °C for 30 min. Finally, cells were analyzed by flow cytometry at an excitation wavelength of 488 nm. For each analysis, 10^4^ events were monitored. The percentage of cells in the sub-G1, G1, S, and G2/M phase were analyzed by a computer (Flowjo 7.6.1 software, TreeStar Inc., Ashland, OR, USA).

### 4.9. Cytokine IL-8 Secretion of Caco-2 Cells after the Short-Term Exposure to Ag NPs

The cytokine IL-8 secreted in the culture medium was measured using the ELISA method. Caco-2 cells (1.2 × 10^5^ per well) were plated in six-well plates and incubated overnight. Then cells were exposed to Ag NPs for 24 h. After that, culture medium was taken out and centrifuged at 2000 rpm for 10 min. The supernatant was collected for IL-8 secretion analysis following the manufacturer’s instructions (Human IL-8 ELISA Kit, Neobioscience Technology Co., Ltd., Shenzhen, China). Results are expressed as pg/mL.

### 4.10. Proliferation of Caco-2 Cells after the Long-Term Exposure to Ag NPs

We investigated the effects of Ag-CIT and Ag-PVP on Caco-2 cells after four exposure cycles. One exposure cycle consists of cell plating, cell attachment (24 h) and Ag NPs exposure for four consecutive days. In detail, cells with a density that could reach confluence after five days were plated in 24-well plates (1 × 10^4^ per well). After one day for firmly attaching cells onto plates, cells were exposed to Ag-CIT or Ag-PVP for 48 h. Then cells were washed with HBSS and medium was replaced by the fresh culture medium containing Ag-CIT and Ag-PVP for another 48-h exposure. After that, cells were then detached from the culture plate with trypsin, counted, and seeded into new 24-well plates (1 × 10^4^ per well) for another exposure cycle.

After each Ag NPs exposure cycle, cells were collected and counted using a blood cell counting plate under an optical microscope after detaching them from plates. Each well was counted three times. Triplet samples were tested for each sample.

### 4.11. Viability, Membrane Integrity, and Live/Dead Cell Staining Assays of Caco-2 Cells after the Long-Term Exposure to Ag NPs

After the third exposure cycle, detached cells were counted and re-plated into 24-well plates (1 × 10^4^ per well) for counting at the end of the forth cycle, 96-well plates (800 per well) for the CCK-8, LDH, live/dead and nuclei staining assays, or 25 cm^2^ flasks (1 × 10^5^ per flask) for IL-8 measured. Then cells were exposed to Ag NPs for the last exposure cycle as described above.

At the end of the forth exposure cycle, the viability, membrane integrity and the live/dead imaging were performed using the CCK-8 kit, LDH kit and live/dead staining kit as described above, respectively. The IL-8 assay is described below.

### 4.12. Nuclei Staining of Caco-2 Cells after the Long-Term Exposure to Ag NPs

At the end of the forth exposure cycle, cells plated in 96-well plates were washed twice by HBSS and then soaked in methanol containing 7% DAPI (4’,6-diamidino-2-phenylindole, Beyotime Institute of Biotechnology, Nanjing, China) at 37 °C for 15 min. After being washed twice with methanol, cells were recorded under a microscope (DMI3000, Leica, Solms, Germany).

### 4.13. Cytokine IL-8 Secretion of Caco-2 Cells after the Long-Term Exposure to Ag NPs

The cytokine IL-8 secreted in the culture medium was measured using the ELISA method. At the end of the forth exposure cycle, culture medium was taken out and centrifuged at 2000 rpm for 10 min. The supernatant was collected for IL-8 secretion analysis following the manufacturer’s instructions (Human IL-8 ELISA Kit, Neobioscience Technology Co., Ltd., Shenzhen, China). Results are expressed as pg/mL.

### 4.14. Statistical Analysis

All means were calculated from at least three independent experiments, and are expressed as the mean ± standard deviation (SD). Analysis of statistical significance was done using the Student’s *t*-test. The results were considered significant if *p* < 0.05.

## 5. Conclusions

In this study, we evaluated and compared the toxicity of three Ag NPs with different surface coatings to Caco-2 cells after 1-day and 21-day exposures to reflect the long-term and low-dose exposure of humans to Ag NPs. Firstly, we found that surface coating affected the toxicity of Ag NPs under both situations. Ag-CIT displays the highest toxicity, while Ag-PVP shows the lowest toxicity. Secondly, the cell proliferation inhibition was observed under both exposure conditions; however, serious cell death was only found after the short-term exposure. Compared with the short-term exposure, the long-term exposure induced the inflammation response before the more serious toxic effects. For the long-term exposure of Caco-2 cells to Ag NPs, 0.3 μg/mL is the non-toxic dose. Finally, we found that the dissolved Ag from Ag NPs contributed less to the toxicity. The ROS generation and subsequent biological responses, play a key role in the cytotoxicity of Ag NPs.

## Figures and Tables

**Table 1:** Summary of average hydrodynamic size and ζ-potential of three Ag NPs in water and full cell culture medium. Ag NPs suspended in medium were kept in the incubator (37 °C, 95% air/5% CO_2_) and the pH value of medium kept around 7.4 during the whole incubation process. All data are represented as the mean ± SD (*n* = 3).

Sample	Time (h)	Ag-B		Ag-CIT		Ag-PVP	
Conditions		DLS (nm)	ζ-potential (mV)	DLS (nm)	ζ-potential (mV)	DLS (nm)	ζ-potential (mV)
Ultrapure water (Milli Q)	0	58 ± 0.5	−12.5 ± 0.6	58 ± 0.5	−5.2 ± 0.4	79 ± 0.5	−16.2 ± 0.4
	24	58 ± 2.2	−13.2 ± 0.3	57 ± 1.0	−15 ± 3.2	81 ± 0.4	−14 ± 1.1
DMEM, 10% FBS	0	50 ± 0.1	−6.2 ± 1.8	51 ± 0.9	−7.7 ± 1.9	70 ± 0.6	−3.4 ± 1.3
	24	62 ± 2.0	−7.7 ± 1.4	54 ± 0.3	−9.6 ± 1.5	63 ± 0.1	−8.4 ± 0.7
	48	137 ± 5.7	−8.8 ± 0.4	138 ± 20	−10.4 ± 1.0	93 ± 2.1	−9.2 ± 0.6

## Supplementary Materials

Supplementary materials can be found at http://www.mdpi.com/1422-0067/17/6/974/s1.
