# Supplementary material for: Toxicological Effects of Caco-2 Cells Following Short-Term and Long-Term Exposure to Ag Nanoparticles
Source: Int J Mol Sci. 2016 Jun 21;17(6):974. doi: 10.3390/ijms17060974 (PMC4926506; doi:10.3390/ijms17060974)
Supplement: Supplementary file 1 [file ijms-17-00974-s001.pdf]

Supplementary Materials: Toxicological Effects of Caco-2 Cells Following Short-Term and Long-Term Exposure to Ag Nanoparticles

Ni Chen, Zheng-Mei Song, Huan Tang, Wen-Song Xi, Aoneng Cao, Yuanfang Liu and Haifang Wang

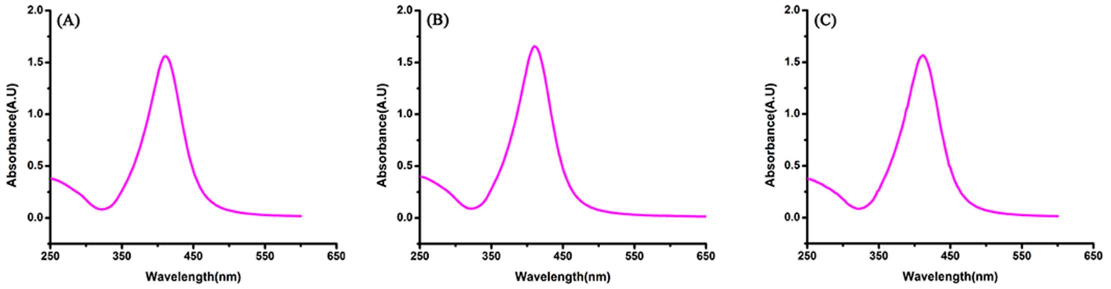

Figure S1. UV-VIS spectra of Ag-CIT (**A**); Ag-B (**B**) and Ag-PVP (**C**). The concentration of three Ag NPs is 12.5 μg/mL.

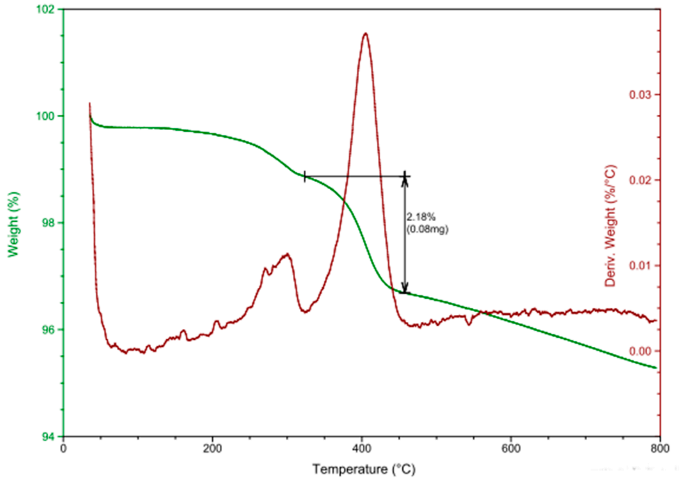

Figure S2. TGA thermograms of the synthesized Ag-PVP.

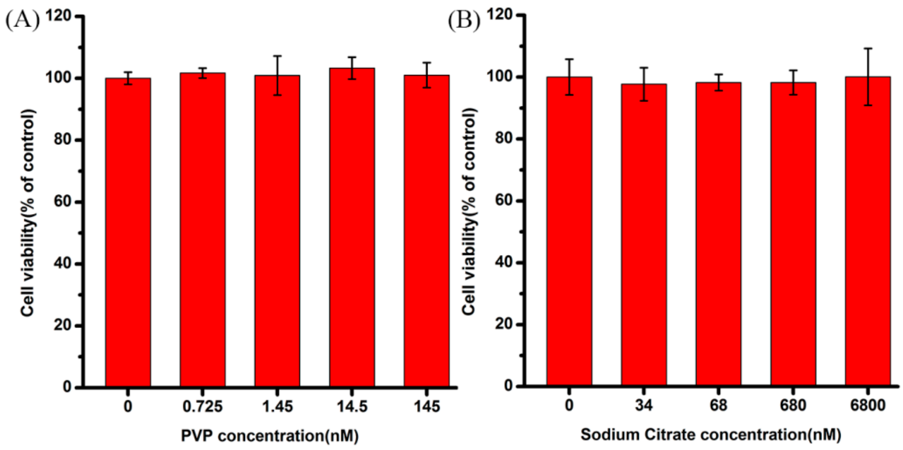

Figure S3. The viability assay of Caco-2 cells after being exposed to PVP (**A**) and sodium citrate (**B**) with different concentrations for 24 h. All data are represented as the mean ± SD (n = 6). $p < 0.05$ comparing with the 0 nM control.

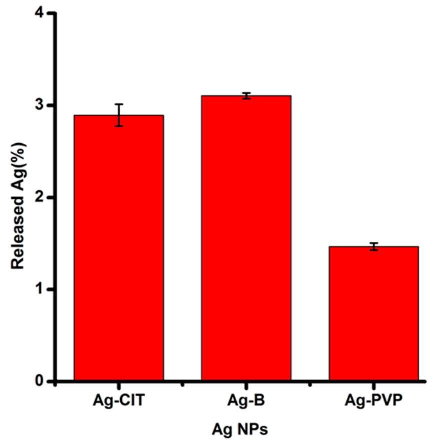

Figure S4. The released Ag from three Ag NPs in culture media. All data are represented as the mean ± SD ($n = 3$).

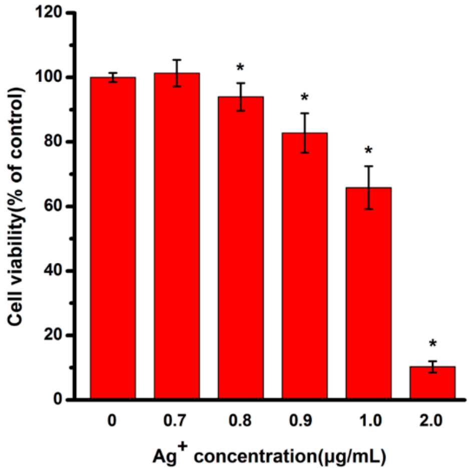

Figure S5. The viability assay of Caco-2 cells after being exposed to silver ions for 24 h. All data are represented as the mean ± SD ($n = 6$). * $p < 0.05$ comparing with the 0 μg/mL control.

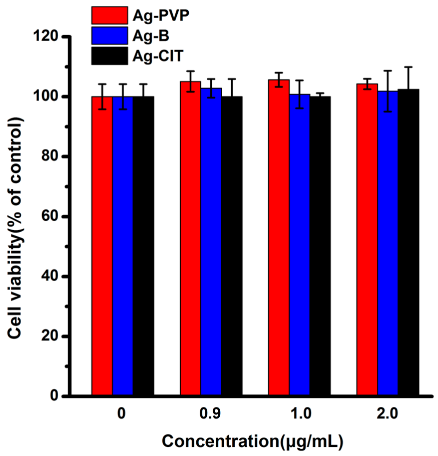

Figure S6. The viability assay of Caco-2 cells after being exposed to the supernatant containing dissolved Ag from Ag NPs for 24 h. All data are represented as the mean ± SD ($n = 6$). $p < 0.05$ comparing with the 0 μg/mL control.
